# Mycotoxin occurrence in kernels and straws of wheat, barley, and tritordeum

**DOI:** 10.1007/s12550-024-00521-w

**Published:** 2024-01-18

**Authors:** Marco Gozzi, Massimo Blandino, Renato Bruni, Luca Capo, Laura Righetti, Chiara Dall’Asta

**Affiliations:** 1 Department of Food and Drug, University of Parma, Parco Area Delle Scienze 27/a, 43100 Parma, Italy; 2 Department of Agricultural Forest and Food Sciences, University of Turin, Largo Paolo Braccini 2, 10095 Grugliasco, Italy; 3 Laboratory of Organic Chemistry, Wageningen University, 6708 WE Wageningen, The Netherlands; 4 Wageningen Food Safety Research, Wageningen University & Research, P.O. Box 230, 6700 AE Wageningen, The Netherlands

**Keywords:** Fusarium, Straw, Tritordeum, Deoxynivalenol, UHPLC-MS/MS

## Abstract

Thirty-two varieties of common and durum wheat, hordeum, barley, and tritordeum collected over two harvesting years (2020 and 2021) were investigated for the presence of multiple *Fusarium*-related mycotoxins in asymptomatic plants. DON, 3-AcDON, 15-AcDON, T-2, HT-2, and ZEN together with the emerging mycotoxin ENN B and the major modified form of DON, namely DON3Glc, were quantified by means of UHPLC-MS/MS. Overall, DON and ENN B were the most frequently detected mycotoxins, albeit large inter-year variability was observed and related to different climate and weather conditions. Straws had higher mycotoxin contents than kernels and regarding DON occurrence tritordeum was found to be the most contaminated group on average for both harvesting years, while barley was the less contaminated one. Emerging mycotoxin ENN B showed comparable contents in kernels compared to straw, with a ratio close to 1 for tritordeum and barley. Regarding the occurrence of the other evaluated mycotoxins, T-2 and HT-2 toxins have been spotted in a few tritordeum samples, while ZEN has been frequently found only in straw from the harvesting year 2020. The data collected confirms the occurrence of multiple *Fusarium* mycotoxins in straws also from asymptomatic plants, highlighting concerns related to feed safety and animal health. The susceptibility of Tritordeum, hereby reported for the first time, suggests that careful measures in terms of monitoring, breeding, and cultural choices should be applied when dealing with his emerging crop.

**Supplementary Information:**

The online version contains supplementary material available at 10.1007/s12550-024-00521-w.

## Introduction

Fusarium head blight (FHB) is a major fungal disease in cereals, caused by multiple *Fusarium* species that commonly co-occur, being *Fusarium graminearum*, *F. culmorum*, *F. poae*, and *F. avenaceum* the most common in Europe (Karlsson et al. 2021).

*Fusarium* species are able to infect at flowering several small-grain cereals such as common wheat (*Triticum aestivum* spp. *aestivum* L.), durum wheat (*Triticum turgidum* spp. *durum* Desf.) and barley (*Hordeum vulgare* L.) (Karlsson et al. 2021), as well as crops obtained from artificial hybridizations of durum wheat such as triticale (× *Triticosecale* Witt., cross with rye) and tritordeum (x *Tritordeum martinii* A. Pujadas, nothosp. nov., cross with a wild barley species, *H. chilense* Roem. et Schultz) (Miedaner et al. 2016; Schöneberg et al. 2018; Spaggiari et al. 2019). Among reported cereals, durum wheat is generally more prone to FHB disease than common one due to genetic and morphological traits (Giancaspro et al. 2018), although a wide varietal susceptibility is reported, particularly within the common wheat genotypes. Although only a few studies are available, recent investigations suggested a high FHB susceptibility in the first commercial cultivars of tritordeum, with a phytosanitary risk in production situations prone to the disease, similar to durum wheat (Landolfi and Blandino 2023). In addition to the genetic background of the host plant, FHB is influenced by agricultural practices which have a direct influence on inoculum production (Scala et al. 2016; Ferrigo et al. 2016). In particular, minimum or reduced tillage and crop rotation with maize or sorghum is reported to increase the potential of FHB epidemics on small-grain cereals (Scarpino and Blandino 2021). The incidence of FHB is also highly correlated with weather patterns and in particular with rainy days with warm temperature at anthesis (Alisaac and Mahlein 2023). Regarding that, climate change impact the weather patterns which in turn are able to deeply affect growth, distribution, and mycotoxin production of pathogenic fungi increasing concerns about food and feed safety (Moretti et al. 2019). In addition, changes in atmospheric CO_2_ concentration and water availability can also modulate plant-pathogen interactions (Scala et al. 2016; Timmusk et al. 2020). Moreover, plant organ damage due to insect pests can favor fungal infection and spreading (Tola and Kebede 2016). Finally, pathogenic *Fusaria* also interact with environmental microbiota giving rise to complex ecological interactions that affect the disease development and mycotoxin production (Venkatesh and Keller 2019; Karlsson et al. 2021).

*Fusarium* diseases cause significant losses in grain yield and affect crop quality, leading also to the contamination by several mycotoxins that can cause a variety of adverse effects to humans and livestock. Occurrence of *Fusarium* mycotoxins in wheat kernels are reported to be positively correlated to FHB disease incidence and severity (Moretti et al. 2019). *Fusarium* mycotoxins include, among others, trichothecenes such as deoxynivalenol (DON), T-2 and HT-2, zearalenone (ZEN), and emerging mycotoxins such as enniatins (ENNs) (Lindblad et al. 2013). Mycotoxins frequently co-occur because of the ability of a single *Fusarium* strain to produce different mycotoxins and to the frequent co-infection by different *Fusarium* species of the same crop grown in open field (Lindblad et al. 2013). Co-occurrence poses a significant threat to public health, due to the additive or synergistic effects of these compounds making a multi-mycotoxin analytical approach of primary importance (Lee and Ryu 2017). In addition, plants possess the ability to biotransform mycotoxins to modified forms that represent an additional potential source of exposure for consumers. For instance, DON is transformed by adding a glucose moiety giving rise to DON3Glc, the most common, and abundant masked mycotoxin reported so far (Berthiller et al. 2013).

While *Fusarium* infection usually occur in kernels, where it induces a high mycotoxin content, these compounds and their plant metabolites have been reported also in spindles, stems, leaves, and straws of cereals (Häggblom and Nordkvist 2015; Moretti et al. 2014; Ulrich et al. 2021). In general, contamination in straws is higher than in corresponding kernels, posing therefore serious threat to animal welfare due to its use as feed (Ulrich et al. 2021). In addition, Brinkmayer et al. (2006), while reporting on the kinetic of DON accumulation in spindles, stems as straws, observed a decrease in concentration after a maximum, probably due to DON biotransformation in plant. Although DON translocation was described by several authors (Kang and Buchenauer 1999; Snijders 2004), it is still unclear at which extent DON and DON3Glc occurrence in kernels and straws may be related to translocation. Furthermore, little is known so far about emerging *Fusarium* compounds such as ENNs, although their occurrence in straws may also pose risk for farming animals. It was indeed reported on the potential accumulation of lipophilic mycotoxins such as ENNs in some animal tissues (Křížová et al. 2021).

This study aimed to carry out a comparison between multiple small-grain cereal genotypes, referring to common and durum wheat, barley, and tritordeum with regard to their susceptibility to multi-mycotoxin accumulation in kernels and straw. Considering the large spectrum of factors that have an influence on infection, spreading and mycotoxin production of *Fusaria* in open field, kernel, and straw samples from varieties grown over two consecutive harvesting years at the same location, were considered in this study. To properly compare the different crops and variety, consistent agronomic conditions were applied, and climate data were recorded.

## Materials and methods

### Chemicals

Analytical standards of DON and its acetylated forms 3-AcDON and 15-AcDON (100 mg/L in acetonitrile), DON3Glc (50 mg/L in acetonitrile), T-2 toxin (100 mg/L in acetonitrile), HT-2 toxin (100 mg/L in acetonitrile), ZEN (100 mg/L in acetonitrile), and ENN B (1 g/L in methanol) were purchased from Romer Labs (Getzersdorf, Austria). UHPLC-grade methanol, acetonitrile, acetic acid, and water were purchased from VWR Chemicals (Radnor, USA). Ammonium acetate was purchased from Sigma-Aldrich (St. Louis, USA).

### Samples and design of the field experiment

Samples from thirty-two varieties, as summarized in supplementary Table S1, grown in a sandy-loam soil at Cigliano (NW Italy, 45°18′N, 8°01′E; altitude 237 m) were collected over two harvesting years (2020 and 2021). These varieties belong to different cereal species, namely common and durum wheat, barley, and tritordeum. For each sample, two technical replicates, obtained from the repetition of the extraction step of samples from the same plots, were measured and results were averaged. Three biological replicates, obtained from different plots for each variety and each year, were analyzed (*N* = 3 × 32 × 2 = 192) for both kernel and straw.

The growing area is characterized by a humid subtropical climate according to the Köppen climate classification and by a probable high FHB pressure, due to the environmental and agronomic conditions involving a frequent rotation with maize (Beck et al. 2018). The daily temperatures and precipitation, shown in Table 1, were measured at a meteorological station near the experimental area.

**Table 1:** Monthly rainfall and growing degree days (GDD) for each growing season

	**2019–2020**		**2020–2021**	
	**Rainfall**	**GDD**^**1**^	**Rainfall**	**GDD**^**1**^
**Month**	(mm)	(Σ °C d^−1^)	(mm)	(Σ °C d^−1^)
November	314	249	4	277
December	132	193	79	144
January	5	168	116	128
February	1	229	29	203
March	62	285	8	286
April	81	414	37	353
May	122	579	69	501
June	113	624	86	674
Nov–June	830	2741	428	2567

^1^Accumulated growing degree days for each month using a 0 °C base value. Source: Rete Agrometeorologica del Piemonte – Regione Piemonte—Assessorato Agricoltura—Settore Fitosanitario, sezione di Agrometeorologia

The agronomic growing technique commonly adopted in the area for small cereal was applied to all the varieties. Briefly, the previous crop was maize, the field was ploughed each year, incorporating the debris into the soil, and this was followed by disk harrowing to prepare a suitable seedbed. Planting was conducted in 12-cm-wide rows at a seeding rate of 450 seeds/m^2^ in November. A total of 130 kg N/ha was applied, split 50 at wheat tillering [growth stage (GS) 23] and 80 kg N/ha at the beginning of stem elongation (GS31), as an ammonium nitrate fertilizer.

A mixture of a strobilurin and a carboxamide fungicide (pyraclostrobin 150 g/ha and fluxapyroxad 75 g/ha, Priaxor^®^, BASF Agricultural Solutions) was applied at the booting stage (GS 39–45) to control foliar diseases. No fungicide was applied at flowering (GS61-65) to control FHB infection. The sowing and harvest dates are reported in supplementary Table S2, together with the dates of the main agronomic practices management, for each growing season. Treatments were assigned to an experimental unit using a completely randomized block design with three replicates. The plots measured 7 × 1.5 m and the grain yields were obtained by harvesting the whole plot using a Walter Wintersteiger cereal plot combine-harvester. The harvested kernels were mixed thoroughly, and 3-kg kernel samples were taken from each plot and were ground to whole-meal using a centrifugal mill equipped with a 1-mm sieve (ETM mill, Vercella Fabio, Mercenasco, Italy) to analyze the mycotoxin content. A straw sample of approximately 5 kg was collected behind the combine-harvester, dried at 60 °C for 72 h in order to reduce the moisture content to 10%, and milled using a centrifugal mill equipped with a 1-mm sieve (ETM mill) to analyze the mycotoxin content.

### Sample preparation

Kernel and straw-milled samples were extracted according to Malachová et al. (2014) with some modifications as follows. Eight milliliters of acetonitrile/water (80:20, v/v) mixture acidified with 0.1% formic acid was added to 2 g of ground kernels (0.5 g of ground straw). The samples were extracted for 90 min using a platform shaker (Ika Werke, Germany) at a speed of 200 strokes/min and subsequently centrifugated for 2 min at 1800 × *g* at room temperature. One thousand microliters of supernatant was transferred into amber glass vials and then injected into the UHPLC-MS/MS system.

### UHPLC-MS/MS analysis

Samples were analyzed according to Righetti et al. (2019) and Malachova et al. (2014), using a UHPLC Dionex Ultimate 3000 coupled to a triple quadrupole mass spectrometer TSQ Vantage (Thermo Fisher Scientific, Waltham, USA) equipped with an electrospray source (ESI). Briefly, the chromatographic separation was obtained using a Sunshell C18 column (Chromanik Technologies, Osaka, Japan) 2.1 × 100 mm, 2.6 μm particle size, heated to 40 °C. 2 μL of sample extract was injected into the UHPLC system and the flow rate was set up to 0.35 mL/min. Gradient elution was performed by using water (eluent A) and methanol (eluent B) both acidified with 0.2% v/v acetic acid. Ammonium acetate was added to the eluent A at the final concentration of 5 mM. Initial conditions were set at 98% A and 2% B for 2 min, then eluent B was increased to 20%, after an isocratic step (6 min), eluent B was further increased to 90%, and this condition was maintained for 10 min until the return to the initial condition. The total run time was 26.5 min. Mass spectrometric analysis was performed both in positive and negative ionization mode in multiple reaction monitoring (MRM), spray voltage 3000 V, capillary temperature 270 °C, vaporizer temperature 200 °C, sheath gas pressure 50 units, and auxiliary gas pressure 5 units. The following quantifier transitions were measured: DON *m/z* 355 > 295 (CE 13 eV) and *m/z* 355 > 265 (CE 19 eV), DON3Glc *m/z* 517 > 457 (CE 17 eV), and *m/z* 517 > 427 (CE 24 eV); 3-AcDON and 15-AcDON *m/z* 397 > 307 (CE 18 eV) and *m/z* 397 > 59 (CE 20 eV); T-2 m*/z* 484 > 215 (CE 19 eV) and *m/z* 484 > 185 (CE 22 eV); HT-2 m*/z* 442 > 263 (CE 11 eV), ZEN *m/z* 317 > 175 (CE 26 eV), and *m/z* 317 > 131 (CE 32 eV); ENN B *m/z* 640 > 528 (CE 20 eV), *m/z* 640 > 314 (CE 31 eV), *m/z* 640 > 214 (CE 26 eV), *m/z* 640 > 196 (CE 29 eV), *m/z* 640 > 186 (CE 37 eV), and *m/z* 640 > 86 (CE 44 eV). Matrix-matched calibration curves (range 1 ng/g–5000 ng/g) showed good linearity for all the target analytes (*R*^2^ > 0.99). Quality control data are reported in our previous works (Righetti et al. 2019; Camardo Leggieri et al. 2020). Data acquisition was performed by Thermo Xcalibur 2.2 software (Thermo Fisher Scientific, Waltham, USA).

### Statistical analysis

ANOVA followed by Tuckey post hoc test (*α* = 0.05) and Pearson’s correlation test (*α* = 0.01) were performed using IBM SPSS v.25.0 (SPSS Italia, Bologna, Italy). Data were log-normalized prior to statistical analysis and the value corresponding to the limit of detection (LOD) was assigned to all samples with levels below this value. Three biological replicates were considered for each variety, and each year, the average of two independent technical replicates were considered for each biological replicate.

## Results and discussion

The two growing seasons showed different meteorological trends as far as both rainfall and temperature (expressed as growing degree days, GDDs) are concerned (Table 1). During the year 2020, the rainfall was much higher after sowing (November) and between stem elongation and flowering (April–May) (Table 1). Overall, within the growing season, rainfall was almost double in 2020 compared to 2021 (830 mm vs 428 mm). 2020 had also a higher temperature than 2019, as described by differences in GDD data (2741 vs 2567 Σ °C d^−1^).

To carry out a comparison between cereals, the thirty-two varieties evaluated in this study were grouped based on the crops to which they belong (supplementary Table S1) and screened for some of the most relevant *Fusarium* mycotoxins by means of UHPLC-MS/MS. DON, 3-AcDON, 15-AcDON, T-2, HT-2, and ZEN together with the emerging mycotoxin ENN B and the major modified form of DON, namely DON3Glc, were quantified as shown in Table 2.

**Table 2:** Overall occurrence of mycotoxins in samples of kernel and straw belonging to the different groups considered in this study. Data are expressed in microgram per kilogram as a range of concentration. The range is given in brackets. LOD: DON and DON3Glc 10 ng/g, 3-AcDON and 15-AcDON 250 ng/g, HT-2 5 ng/g, T-2, ZEN and ENN B 1 ng/g. Number of samples (*N*): common wheat (*N* = 300), durum wheat (*N* = 24), barley (*N* = 24), tritordeum (*N* = 36)

Mycotoxins	Crops	Kernels		Straws	
		2020	2021	2020	2021
DON	Common wheat	2019 *ab* (360–6253)	147 *ab* (40–472)	4117 *a* (656–12,333)	601 *ab* (60–1644)
	Durum wheat	3487 *ab* (2300–4673)	310 *a* (218–402)	3268 *a* (2139–4395)	521 *a* (377–665)
	Tritordeum	4098 *a* (3854–4466)	335 *a* (299–363)	7232 *a* (5480–8300)	386 *a* (327–427)
	Barley	546 *b* (465–627)	57 *b* (45–69)	805 *b* (904–706)	284 *b* (< 40–407)
DON3Glc	Common wheat	88 *ab* (< LOD-357)	< LOD	1049 (60–3007)	149 (< LOD-440)
	Durum wheat	411 *ab* (190–632)	< LOD	453 (< LOD-896)	< LOD
	Tritordeum	469 *a* (385–534)	< LOD	591 (449–728)	93 (63–125)
	Barley	108 *b* (< LOD-206)	< LOD	377 (355–398)	< LOD
ZEN	Common wheat	24 (< LOD-90)	< LOD	93 (4–328)	< LOD
	Durum wheat	41 (12–70)	< LOD	233 (210–255)	< LOD
	Tritordeum	5 (< LOD-7)	< LOD	138 (54–221)	< LOD
	Barley	12 (< LOD-18)	< LOD	142 (4–318)	< LOD
ENNB	Common wheat	365 *b* (91–1153)	5 *b* (4–18)	671 (263–1379)	41 (< LOD-295)
	Durum wheat	4037 *a* (1011–7063)	8 *b* (4–13)	1238 (1044–1432)	230 (62–398)
	Tritordeum	1757 *a* (1588–1852)	22 *a* (11–34)	1415 (670–2285)	70 (24–132)
	Barley	1110 *a* (1102–1118)	52 *a* (24–80)	1115 (510–1721)	35 (23–47)
T-2	Common wheat	< LOD	< LOD	< LOD—83	< LOD
	Durum wheat	< LOD	< LOD	< LOD—127	< LOD
	Tritordeum	< LOD	5 (4–12)	< LOD—38	< LOD
	Barley	< LOD	< LOD	< LOD—143	< LOD
HT-2	Common wheat	< LOD	< LOD	< LOD	< LOD
	Durum wheat	< LOD	< LOD	< LOD	< LOD
	Tritordeum	< LOD	< LOD	< LOD	< LOD
	Barley	< LOD	< LOD	< LOD	< LOD
3-/15-AcDON	Common wheat	< LOD	< LOD	< LOD	< LOD
	Durum wheat	< LOD	< LOD	< LOD	< LOD
	Tritordeum	< LOD	< LOD	< LOD	< LOD
	Barley	< LOD	< LOD	< LOD	< LOD

Overall, DON and ENN B were the most frequently detected mycotoxins. In particular, samples from the harvesting year 2020 showed a higher contamination level of about an order of magnitude on average for both kernels and straw compared to the harvesting year 2021 (Table 2). However, such a large difference was not surprising given the different meteorological scenario previously described. In addition, straw was found to be on average more contaminated than kernels for the vast majority of samples, in accordance with literature data for wheat (Ji et al. 2015).

**Fig. 1:**
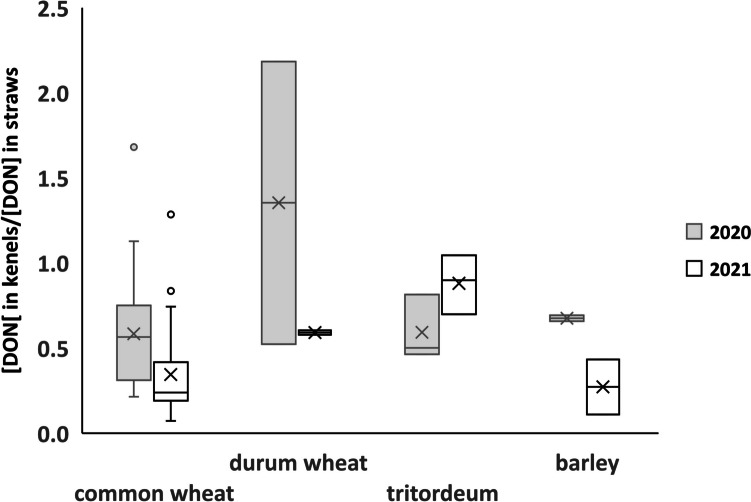
Ratios of DON contamination in kernels and straws per crops and harvest year (light gray: 2020; white: 2021)

Regarding DON occurrence in kernels, significant differences were observed. Tritordeum was found to be the most contaminated group on average for both the harvesting years (4098 ± 324 ng/g for 2020 and 335 ± 33 ng/g for 2021), while barley was the less contaminated one (546 ± 114 ng/g for 2020 and 57 ± 17 ng/g for 2021). However, according to one-way ANOVA, the only significant difference found was between tritordeum and barley (2020: *p* = 0.012; 2021: *p* = 0.021). About DON occurrence in straw, tritordeum was found as the most contaminated group on average for 2020 (7232 ± 1529 ng/g) and common wheat the most contaminated one for 2021 (601 ± 453 ng/g). According to one-way ANOVA (*α* = 0.05), the only significant difference found for straw (year 2020) was between tritordeum and barley (*p* = 0.010). In addition, according to Pearson’s correlation test, a significant positive correlation was found between DON occurrence in kernels and straw for the harvesting year 2020 (*r* = 0.568, *p* < 0.001) and 2021 (*r* = 0.285, *p* = 0.019) when all varieties were considered, consistently with what was reported in the literature for wheat, that was the most represented crop in this dataset (Bissonnette et al. 2018a). The ratio between DON content in kernels and straws is represented in Fig. 1. As shown, although DON mean content is higher in straws than in kernels over both harvesting years and across all species, the ratio is not fully conserved. Overall, our results are consistent with those previously reported (Haggblom and Nordkvist, 2015; Nordkvist and Haggblom 2014; Brinkmeyer et al. 2006; Daenicke et al. 2006), all pointing out a higher DON content in straws than in kernels.

Unfortunately, our findings cannot explain the route of DON contamination in straws. Literature evidence is controversial, with studies reporting on *F. graminearum* colonization in straws deriving from fungal inocula present in soil and/or seeds and other discussing potential fungal infection at the flowering stage (Ludewig et al. 2005; Wegulo et al. 2012; Moretti et al. 2014). In our study, no disease symptoms were visually observed in the field. This is also in agreement with previous findings. In particular, Haggblom and Nordkvist (2015) pinpointed the high DON level recorded despite the good agricultural practices applied and the lack of visual symptoms of fungal disease observed over the harvest season.

Due to its polarity, also a translocation of DON from the apical parts of the plant cannot be ruled out, following fungal infection at the flowering stage. However, considering the higher content of DON in straws compared to kernels, in our case, such occurrence is unlikely to be ascribed to translocation only.

DON3Glc was found below the limit of quantification for the majority of samples from 2021. When the harvesting year 2020 was considered, a strong positive correlation with DON occurrence was found for both kernels (*r* = 0.678, *p* < 0.001) and straw (*r* = 0.582, *p* < 0.001) consistently to what was reported by Ovando-Martínez et al. (2013). In 2020, the DON3Glc/DON ratio lays between 0.04 and 0.20 in kernels and 0.08 and 0.47 in straws, being the highest ratio found in barley (0.20 and 0.47, respectively). This is consistent with the scientific literature reporting a biotransformation of about 20–30% for cereal grains (Moretti et al. 2014). Based on the collected data, the ratio appears slightly higher in straws than in kernels, suggesting a higher accumulation of DON3Glc in straws. This may be ascribed to a tissue-dependent biotransformation, as already reported by Righetti et al. (2020) or to DON3Glc translocation from kernels. Due to the higher polarity of the glucoside compared to the aglycone (logP =  − 2.3 and − 0.7, respectively, based on PubChem records), a higher translocation efficiency is plausible, although more dedicated studies are needed to fully explore this hypothesis. 

While observing the contamination by ENN B, this mycotoxin was frequently spotted below the limit of quantification for the year 2021. When the harvesting year 2020 was considered, common wheat was found as the less contaminated group for kernels (365 ± 222 ng/g) and significantly differs (*p* < 0.001) from the other groups according to one-way ANOVA, while for straw no significant differences emerged between groups. Differently from what observed for DON, ENN B content in kernels and straws is comparable, with a ratio close to 1 for tritordeum and barley. The higher lipophilicity of ENN B (logP = 6.5 based on PubChem records) compared to DON and DON3Glc makes plant translocation less likely.

Regarding the occurrence of the other evaluated mycotoxins, T-2 and HT-2 toxins have been spotted in few tritordeum samples, while ZEN has been frequently found only in straw from the harvesting year 2020. This is also in line with what was previously reported (Haggblom and Nordkvist 2015). Finally, acetylated forms of DON have been not detected at all.

The higher DON contamination reported in this study for durum wheat and tritordeum kernels compared to common wheat and barley was observed despite the great difference in contamination level between the harvesting years considered. This aspect needs to be carefully evaluated in light of the frequent multiple infection of *Fusarium* species that take place in the field and the consequent co-occurrence of *Fusarium* mycotoxins, some of which are not regulated nor routinary evaluated. Particularly, these findings underline the need for increased attention to the safety profile of tritordeum, considering its current use as an alternative to produce food products. Therefore, although further investigations involving a larger set of tritordeum varieties seem to be warranted to consolidate these results, the choice of the growing areas for the cultivation of this new crop need to consider carefully the potential constraints related to phytosanitary risk. Moreover, future tritordeum breeding will require the use of durum parental lines specifically selected for the FHB resistance.

Ultimately, this study confirms the high levels of *Fusarium* mycotoxins in straws from asymptomatic plants, highlighting that multiple different noxious compounds co-occur. From a feed safety and animal health perspective, this can pose a risk, especially for monogastric livestock (Bissonnette et al. 2018b). In addition, from an agronomic point of view, the source of such contamination is still unclear and worth of further investigation. In particular, the potential translocation of DON and DON3Glc in the plant as well as the tissue-specific biotransformation may provide useful insights into plant resistance mechanisms for biocontrol strategies development.

### Supplementary Information

Below is the link to the electronic supplementary material.

Supplementary file1 (DOCX 15 KB)

## Data Availability

Not applicable.
