# Supplementary material for: Mycotoxin occurrence in kernels and straws of wheat, barley, and tritordeum
Source: Mycotoxin Res. 2024 Jan 18;40(1):203–10. doi: 10.1007/s12550-024-00521-w (PMC10834653; doi:10.1007/s12550-024-00521-w)
Supplement: Supplementary file 1 — Supplementary file1 (DOCX 15 KB) [file 12550_2024_521_MOESM1_ESM.docx]

**Supplementary material**

**Table S1.** Varieties considered in this study and corresponding species or hybrids.

| **Crop** | **Variety** |
| --- | --- |
| Common wheat | Adriatic; Akim; Altamira; Amburgo; Argone; Arkeos; Artico; Aubusson; Bagou; Bologna; Bramante; Canaletto; Cosmic; Donatello; Ethic; Hystar; Jaguar; Lancillotto; Santorin; Solehio; SY Alteo; SY Capitano; SY Liam; SY Passion; Verna |
| Durum wheat | Antalis; Odisseo |
| Barley | Cometa, Ketos |
| ×Tritordeum | Aucan, Bulel, Coique |

**Table S2**. Main agronomic information of the agronomical management for each growing season.

| **Year** | **Sowing date** | **N fertilization** | | **Fungicide**^1^ | **Harvest date** |
| --- | --- | --- | --- | --- | --- |
|  |  | **GS 23** | **GS 31** | **GS 39-45** |  |
| 2020 | 6/11/2019 | 5/03/2020 | 3/04/2020 | 23/04/2020 | 29/06/2020 |
| 2021 | 3/11/2020 | 2/03/2021 | 8/04/2021 | 21/04/2021 | 5/07/2021 |

^1^from the end of stem elongation (GS39) to the booting stage (GS45), according to the different GS of considered genotypes.
